# Serotonin transporter gene polymorphism modulates inflammatory cytokine responses during acute stress

**DOI:** 10.1038/srep13852

**Published:** 2015-09-09

**Authors:** Kaori Yamakawa, Masahiro Matsunaga, Tokiko Isowa, Hideki Ohira

**Affiliations:** 1 Department of Psychology, Graduate School of Environmental Studies, Nagoya University, Aichi, Japan; 2 Department of Psychology, School of Humanities, Tokaigakuen University, Aichi, Japan; 3 Department of Health and Psychosocial Medicine, School of Medicine, Aichi Medical University, Aichi, Japan; 4 School of Nursing, Faculty of Medicine, Mie University, Mie, Japan

## Abstract

Cytokines are important mediators of various stress-related modulations of immune function. A major genetic factor determining inter-individual differences in stress reactivity is polymorphisms of the serotonin (5-hydroxytryptamine, 5HT) transporter (5HTT) gene. A short (S) variant, compared with a long (L) variant, of the promoter region of the 5HTT gene-linked polymorphic region (5HTTLPR) has been related to emotional and stress hyper-reactivity. The present study examined whether the 5HTTLPR can modulate responses of inflammatory cytokines under acute stress. Nine Japanese male participants carrying two copies of the S alleles and nine Japanese males carrying S and L alleles underwent the Trier Social Stress Test (TSST). Inflammatory cytokines, endocrine parameters, heart rate and subjective stress were measured before, during and after the task. The participants carrying the SS alleles, but not those carrying the SL alleles, showed a significant increase of IL-1β immediately after TSST. This hyper-reactivity to acute stress in individuals with the SS alleles was also observed in their heart rate and cortisol levels. These results suggest that the S allele of the 5HTTLPR is consistently associated with stress reactivity in multi-level stress-related biological systems.

Acute stressors stimulate the sympathetic and adrenomedullary (SAM) system, as well as the hypothalamic–pituitary–adrenal (HPA) axis, leading to physiological responses that are essential for adaptation to one’s environment. Dysfunction in the levels of reactivity can result in physical and psychiatric disorders^1^,^2^. One of the major genetic factors determining inter-individual differences in stress reactivity is the serotonin (5-hydroxytryptamine, 5HT) transporter (5HTT) gene polymorphism, which mediates reuptake and recycling of released serotonin following neuronal stimulation. Transcriptional activity of the L allele of the gene is twice more than that of the S allele^3^. Thus, the S promoter allelic variant is linked to reduced 5HTT mRNA expression, resulting in less serotonin reuptake compared to the L allelic variant^4^,^5^.

Many studies have suggested an association between 5HTT genotype and physiological reactivity to acute stressors. Compared with wild-type controls, 5HTT knockout mice show facilitated catecholamine responses to brief and mild stressors^6^,^7^. Li *et al.*^7^ found that mice with diminished or absent 5HTT gene function exhibit greater elevations of adrenocorticotropic hormone, a typical stress hormone, in response to acute stress than their control littermates. Human studies suggest that 5HTT may modulate the activity of the HPA axis under stressful conditions^8^,^9^. Individuals with the homozygous double S alleles of the 5HTT gene-linked polymorphic region (5HTTLPR) showed greater blood pressure reactivity to an acute stressor compared with the SL allele and LL allele carriers^8^. Moreover, S allele carriers show enhanced cortisol secretion in response to acute psychological stressors^10^.

Previous studies^11^,^12^ have demonstrated that pro-inflammatory cytokines that increase after acute psychological stress play key roles in physiological stress responses. Pro-inflammatory cytokines are defined as cytokines that promote systemic inflammation by transporting immune cells to the site of infection or injury^13^ and play important roles in eliminating invading viruses and bacteria as well as conserving energy in the immune system^14^. Despite the importance of pro-inflammatory cytokines in physiological stress responses, the association between 5HTTLPR and the reactivity of pro-inflammatory cytokines to acute stress situations has rarely been examined. An animal study revealed that the release of glucocorticoids in response to acute stressors is associated with the inhibition of interleukin-6 (IL-6) responses in S carriers^15^. Fredericks *et al.*^16^ found higher IL-6/IL-10 ratios, indicating the balance of pro- and anti-inflammation, in S allele human participants at baseline and during stress. They observed an effect of the 5HTTLPR S allele on pro-inflammatory bias, although no such effects were found in 5HTTLPR on IL-6 and IL-10 absolute values. Because pro-inflammatory cytokines may mediate behavioural, psychological and physiological responses during acute stress situations^17^, it is critical to examine the effects of 5HTTLPR on stress reactivity across multi-level physiological systems including pro-inflammatory cytokines in the same experimental conditions.

The main purpose of this study was to examine whether the 5HTT genotype affects the reactivity of pro-inflammatory cytokines as well as autonomic nervous and endocrine parameters to an acute stressor. We measured concentrations of IL-1β and IL-6 in the peripheral blood to represent typical pro-inflammatory cytokines and the IL-6/IL-10 ratio as representing the balance of pro- and anti-inflammation^16^,^18^,^19^. Additionally, we measured the levels of natural killer (NK) cells in the peripheral blood, which have also been shown to increase in response to acute stress^19^,^20^. The heart rate (HR) and heart rate variability (HRV) served as indices of the SAM system activity whereas the cortisol level acted as an index of HPA axis activity. For this purpose, we used the Trier Social Stress Test^21^, which is a well-defined, standardised acute psychological stress task^22^.

## Method

### Participants

Nine participants with the 5HTTLPR S allele and nine with the SL allele participated in the present study. All participants were male, right-handed, native Japanese, and undergraduate or graduate students of Nagoya University (age (mean ± standard error); SS: 20.78 ± 0.26 years, SL: 21.00 ± 0.25 years). None of the participants was suffering from any chronic illnesses and none were taking medications known to influence immunity. Only men were studied to avoid influences of the menstrual cycle on any physiological stress responses in women^23^. All participants provided their informed consent to participate in the study, in accordance with university policy. The study was approved by the Ethics Committee of Nagoya University.

For determining genotypes, genomic DNA was extracted with a DNA Extractor WB-Rapid Kit (Wako Inc., Osaka, Japan) from frozen blood samples collected from the participants. We analyzed samples using polymerase chain reaction according to previously reported methods, generating L (528 bp) and S (484 bp) fragments^4^. Individuals carrying double copies of the S allele (SS genotype) and individuals carrying the S and L alleles (SL genotype) were identified. We compared stress responses in the SS (n = 9) and SL (n = 9) genotypes, as the proportion of LL genotypes is small in the Asian population^24^.

To evaluate participants’ levels of trait anxiety as a potential confounder, we asked the participants to complete a Japanese version^25^ of the state-Trait Anxiety Inventory (STAI)^26^.

### Biological measures

Blood samples were collected in EDTA tubes to determine changes in the proportion of NK cells among peripheral circulating lymphocytes before and after the TSST. Two-color flow cytometry was performed. The whole-blood lysis method was used to stain the NK cells with a fluorescein isothiocyanate (FITC)-conjugated anti-CD16 antibody (Ab) (DakoCytomation, Carpinteria, CA) as well as a R-phycoerythrin (RPE)-conjugated anti-CD56 Ab (DakoCytomation), as described previously^27^. The samples were analyzed on a FACSCanto II flow cytometer (Becton Dickinson, Franklin Lakes, NJ) using FACSDiva software (Becton Dickinson).

Additional blood samples were collected in EDTA tubes and centrifuged at 3,000 × g for 10 min to determine changes in inflammatory cytokine and cortisol levels in the plasma before and after the TSST; the plasma was then separated and stored at −80 °C until analysis occurred. Cytokine levels (IL-1β, IL-6, and IL-10) in the plasma were determined using a FlowCytomix Assay (Bender Medsystems, Wien, Austria), according to the manufacturer’s instructions. The intra-assay coefficient of variation for each sample was 4.5–12.0%, and inter-assay coefficient of variation for each sample was 3.3–16.3%. The cytokine detection threshold was 4.2 pg/ml. Cortisol plasma concentration was measured using a cortisol ELISA kit (Oxford Biochemical Research Inc., Oxford, MI). Here, the intra-assay coefficient of variation was 3.4–3.7%, and the inter-assay coefficient of variation was 3.8–6.4%. Limit of detection was 0.3 μg/ml.

Cardiodynamic activity was recorded using an electrocardiogram (ECG) at 500 Hz using an MP 100 system (Biopac Systems Inc.) with Ag/AgCL electrodes placed on the extremities. Analysis of ECG waveforms was performed using AcqKnowledge software for MP 100. After rejection of artifacts in the ECG waveforms, HR and inter-beat-interval data were acquired during the baseline period, at three periods during the stress task (0–10, 10–15, and 15–20 min) and at each rest period. The inter-beat-interval data were subsequently analyzed to yield HRV. Data from each participant were subjected to ocular inspection and only completely artifact-free data were used for estimation of the inter-beat-intervals. The inter-beat-interval data were resampled at 4 Hz to obtain equidistant time series values. A power spectrum density was then obtained through a fast Fourier transformation of the tachogram. In connection with the fast Fourier transformation, the data were detrended linearly and filtered through a rectangular window. Power spectrum integral was studied in two major frequency bands, the HF (0.15–0.5 Hz) and LF (0.05–0.15 Hz) components. The former is related to respiratory sinus arrhythmia and is exclusively attributable to parasympathetic influence reflecting vagal activity, and in the latter case, the LF component mirrors the baroreceptor feedback loop that controls blood pressure and appears to reflect both sympathetic and parasympathetic activity. Consequently, the HF component and relative contributions of LF and HF power (LF/HF), which reflect sympathovagal balance, were considered^28^.

### Stress task (the TSST)

The TSST includes a fake speech as well as a mental arithmetic task^21^. This is a standardized procedure for the induction (in laboratory settings) of acute psychological stress associated with the HPA axis and autonomic nervous system arousal^29^. After the participants were introduced to the TSST (1 min), they were given 10 min to prepare themselves for a speech about school life (5 min), followed by a mental arithmetic task in front of an audience (5 min). The participants were told that they would be videotaped for further analysis of their behavior.

### Procedures

The participants were instructed to eat a light breakfast on the morning of the experiment; caffeinated beverages were not allowed. They were also instructed to paste a monoanesthetic seal at the location of the cannula insertion in their arms about 1 h before the experimental sessions to reduce pain. Participants suffering from an infectious illness within 2 weeks of the experiment were rescheduled.

**Figure 1:**
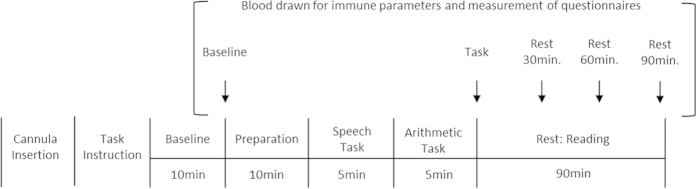
Experimental protocol of the present study.

For minimum confounds in the form of diurnal variations in hormone levels, experimental sessions for both conditions started at the exactly same time between 9:00 a.m. and 15:00 p.m. for each participant. Furthermore, experimental time (morning or afternoon) was counterbalanced between participants. The session was composed of a baseline period, the TSST, and 4 rest periods (Fig. 1). After the participants entered the experiment room, a cannula was inserted into the forearm vein of the non-dominant arm. Next, electrodes for electrocardiographic measurements were attached. After a first rest period of 10 min, the first blood sample was taken as baseline sample, and the participants were asked to fill out a questionnaire. In this questionnaire, the participants were asked to subjectively evaluate stress intensity on visual-analog scales (0–100%) as a psychological measure of subjective stress level. TSST instructions were then provided (speech followed by a simulated interview). Following this, the participants prepared their speech (10 min) and were then exposed to a simulated interview (5 min) conducted by two interviewers in front of a video camera, followed by a mental arithmetic task (5 min). Immediately after the task, a second blood sample was taken and the participants again filled out the questionnaire. Finally, the participants read newspapers during the 90 min rest period. After each rest period (30, 60, and 90 min after the completion of the TSST), the third, fourth, and fifth blood samples were taken and the questionnaire was filled out for a final time. ECG was measured continuously throughout the experimental session. After the end of the procedure, the electrodes and cannula were removed and the participants fully debriefed and thanked.

### Statistical analysis

The present data were analyzed using repeated-measures analyses of variance (ANOVAs) with a between-participants factor of Group (SS genotype vs. SL genotype) and a within-participants factor of Period (at Baseline, Task, and Rest_30_ _min_, Rest_60_ _min_, Rest_90_ _min_), with regard to intensity of stress, cortisol, lymphocyte and cytokine data. Cardiovascular data was also analyzed using repeated-measures ANOVAs with a between-participants factor of Group and a within-participants factor of Period (at Baseline, Task_10_ _min_, Task_15_ _min_, Task_20_ _min_, Rest_30_ _min_, Rest_60_ _min_, Rest_90_ _min_). The Greenhouse–Geisser epsilon correction factor, *ε*^30^, was used where appropriate. In cases where significant interactions were found, post hoc analyzes using Bonferroni tests (*p* < 0.05) were conducted to examine which combinations of data points differed significantly. Effect sizes are presented as η^2^-values.

## Results

The basic characteristics of the participants are summarised in Table 1. Genotype groups did not differ significantly in terms of age, height, weight, BMI or trait anxiety.

Psychological data collected during the stress task and rest periods are presented in Table 2. Repeated measures ANOVAs revealed a statistically significant main effect of the Period [*F* (1.99, 37.83) = 17.70, *p* < 0.05, *η*^2^ = 0.48]. Post hoc analysis (*p* < 0.05) indicated that perceptions of stress after the task were higher than at baseline. However, the interaction between the Group and Period was not statistically significant for the perceptions of stress [*F* (1.99, 37.83) = 0.26, not significant (n.s.), *η*^2^ = 0.14].

**Figure 2:**
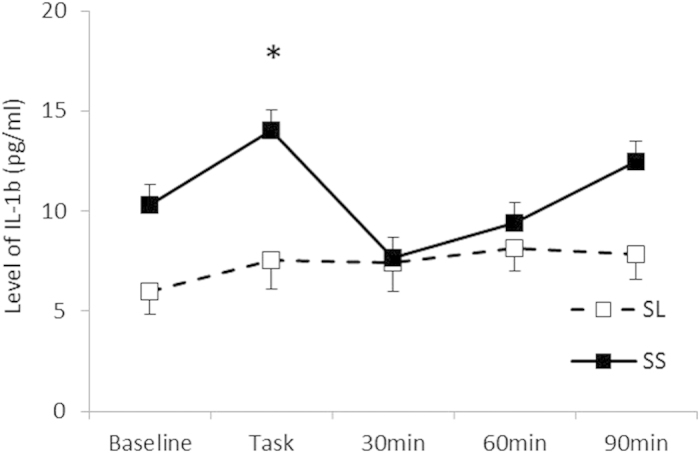
Interleukin-1β levels for each group. Error bars indicate the standard error of the mean. *Significant difference from the baseline value in each group (*p* < 0.05).

**Figure 3:**
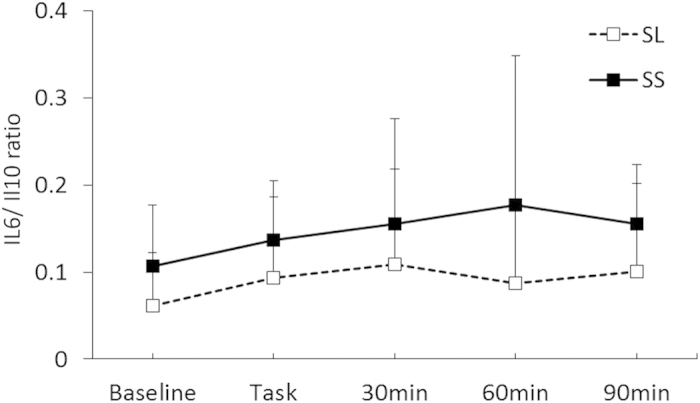
Interleukin (IL)-6/IL-10 ratio for each group. Error bars indicate the standard error of the mean.

**Figure 4:**
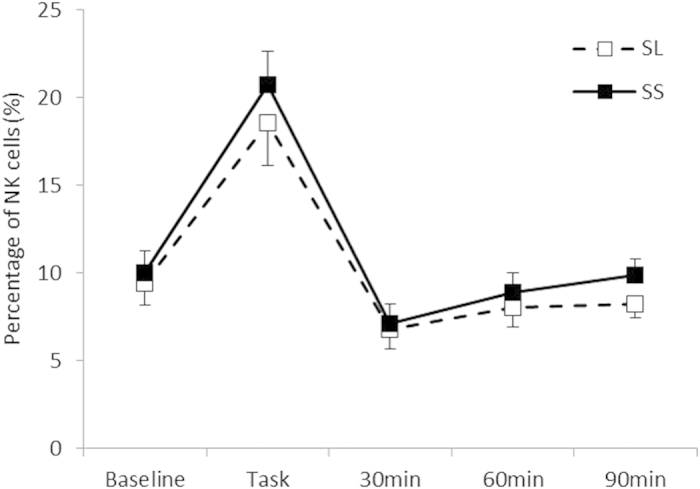
Percentage of natural killer cells for each group. Error bars indicate the standard error of the mean.

**Figure 5:**
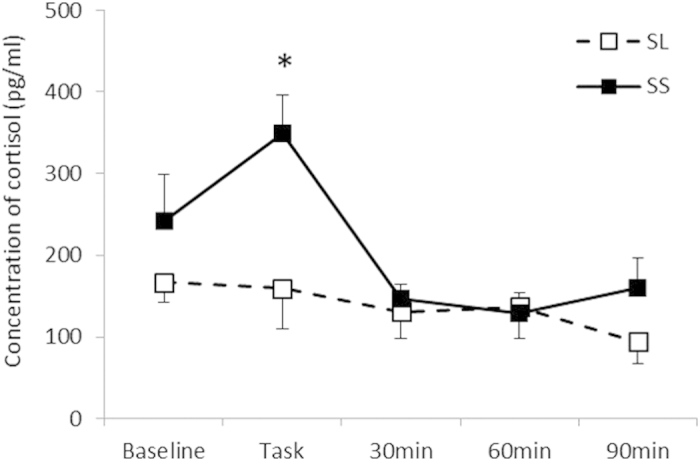
Cortisol concentrations for each group. Error bars indicate the standard error of the mean. *Significant difference from the baseline value in each group (*p* < 0.05).

As shown in Fig. 2, a statistically significant interaction between the Group and Period was found for IL-1β [*F* (4, 60) = 2.77, *p* < 0.05, *η*^2^ = 0.16], and post hoc analysis (*p* < 0.05) indicated that IL-1β levels after the task in the SS group were higher than those in the SL group, although no significant difference was observed during the rest periods. Although the interaction between the Group and Period for IL-6 was not statistically significant [*F* (4, 60) = 0.77, n.s., *η*^2^ = 0.49; Table 3], the main effect of the Group on the IL-6/IL-10 ratio was statistically significant [*F* (1, 19) = 4.89, *p* < 0.05, *η*^2^ = 0.21; Fig. 3], which indicated that the SS group consistently showed a higher IL-6/IL-10 ratio. For a proportion of immune parameters, a significant main effect of Period for CD16 + CD56 + NK cells was observed [*F* (1.74, 27.78) = 55.39, *p* < 0.01, *η*^2^ = 0.77]. Post hoc analyses (*p* < 0.05) indicated that the proportions of NK cells after the stress task were higher than those at the baseline for all groups, although no significant interaction of Group and Period was observed for NK cells [*F* (1.74, 27.78) = 1.21, n.s., *η*^2^ = 0.70; Fig. 4]. In terms of cortisol concentrations, there was a significant interaction between the Group and Period [*F* (4, 60) = 2.55, *p* < 0.05, *η*^2^ = 0.15], as shown in Fig. 5. Cortisol levels significantly increased after the task compared with those at the baseline or during the rest periods only in the SS group. No such effect was found in the SL group (*p < *0.05).

**Figure 6:**
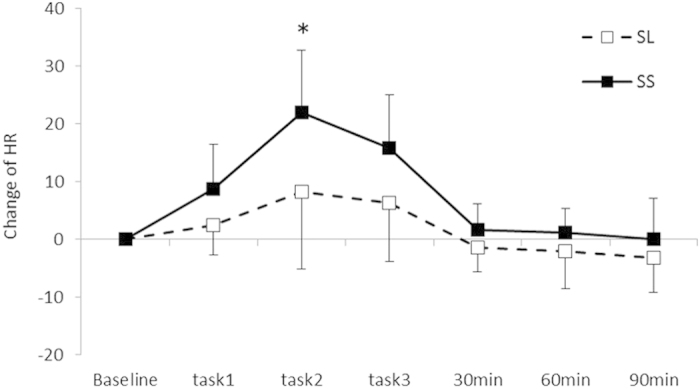
Changes in heart rate throughout the experimental sessions. Error bars indicate the standard error of the mean. *Significant differences from the baseline value for each group (*p* < 0.05).

Cardiovascular data collected during the stress task and rest periods are presented in Fig. 6. A significant interaction was found between the Group and Period for the change in HR [*F* (2.23, 35.75) = 3.29, *p* < 0.05, *η*^2^ = 0.17]. Further analyses (*p* < 0.05) revealed that HR changes in the SS group were greater during the speech tasks as compared with the SL group. Additionally, HR changes were greater in the SS group during the tasks than at baseline or during the rest periods but no such difference was observed in the SL group. Table 4 depicts the high frequency (HF) components and the low frequency (LF)/HF ratio of HRV at baseline and during the task periods (task_10 min_, task_15 min_ and task_20 min_) and rest periods (Rest_30 min,_ Rest_60 min_ and Rest_90 min_). There were no significant main effects or interactions for the HF components or LF/HF ratio [*F* (6, 96) = 1.04, n.s., *η*^2^ = 0.61; *F* (3.44, 55.05) = 1.32, n.s., *η*^2^ = 0.76].

## Discussion

As predicted, the present study demonstrated a pronounced increase in IL-1β levels in 5HTTLPR SS carriers as compared with L carriers. Steptoe^12^ proposed several possible mechanisms that may contribute to acute changes in the levels of circulating pro-inflammatory cytokines, particularly IL-1β, in response to acute stress. One plausible mechanism is that immune cells that synthesise and release pro-inflammatory cytokines are mobilised into the peripheral bloodstream from marginal blood pools by the activation of the sympathetic nervous system^31^,^32^. In line with this theory, acute psychological stress tasks do induce transient elevations of immune cell numbers via the activation of the sympathetic nervous system^20^. Bosch *et al.*^33^ demonstrated an elevation of monocytes, which mainly release IL-1β^34^, in the peripheral blood following the exposure to acute stress. Therefore, the difference in IL-1β reactivity between the 5HTTLPR genotypes observed in the present study can be attributed to a difference in the degree of sympathetic nervous system activation.

Supporting this inference, we observed a greater transient increases in HR after the stress task in SS allele participants than in SL allele participants. Although the effect was not statistically significant, the SS group in this study exhibited increased stress reactivity in the LF/HF ratio, which is an index of relative sympathetic activity. Previous studies have shown that sympathetic nervous system activation that mediates the mobilization of monocytes is greater in S allele carriers than in L allele carriers^35^,^36^. Serotonin neurotransmitter function in S allele carriers may be reduced via increased firing rates of serotonin neurons^37^ and the down-regulation of inhibitory 5HT1A receptors^38^. In addition, Ohira *et al.*^8^ reported that participants carrying the SS allele demonstrated stronger reactivity in terms of blood pressure and the secretion of epinephrine as well as greater activation in stress-related brain regions such as the hypothalamus, which regulates the HPA and SAM axes. Taken together, the present finding of enhanced acute stress reactivity, as indexed by IL-1β in SS carriers, can be interpreted in terms of the enhanced sensitivity of the sympathetic nervous system during acute stress.

Consistent with the findings of Fredrick *et al.*^16^, we found higher IL-6/IL-10 ratios in the SS group at baseline and during stress, which suggests that the smaller amount of serotonin found in the brains of those with the S allele of 5HTTLPR may lead to chronic pro-inflammatory bias. Thus, the present study showed that 5HTTLPR can regulate both the transient reactivity of pro-inflammatory cytokines to acute stress as reflected by IL-1β and the chronic tendency of inflammation as reflected by the IL-6/IL-10 ratio. The effect of 5HTTLPR on the chronic IL-6/IL-10 ratio was consistently supported by our unpublished data with a larger sample size (N = 154: Supplementary figure online), suggesting the reliability of the findings from the present study.

In this study, we did not observe 5HTTLPR effects on stress reactivity in NK cell proportions and IL-6 levels. Many studies have indicated a transient increase in NK cell proportions in response to acute stress^39^,^40^, which is consistent with the results of the present study. Benschop *et al.*^41^ proposed that elevated blood pressure elicited by sympathetic activity may physically transport NK cells into the peripheral blood. Accordingly, Kimura *et al.*^20^ found positive correlations between sympathetic cardiovascular activity and variations in subsets of lymphocytes, including NK cells, in acute stress situations. On the other hand, corticosteroids that are released via the HPA axis selectively inhibit the redistribution of NK cells^42^. Given that the 5HTTLPR S allele was linked with enhancement of the HPA axis (cortisol) activities in the present study, which is consistent with the previous report^9^, it seems reasonable to propose that the effects of 5HTTLPR on the two systems (SAM and HPA) appear to be antagonistic. Such enhancing (SAM) and inhibiting (HPA) effects on NK cells may offset the influences of the 5HTTLPR S allele on NK cell reactivity. Similarly, Zrkovic *et al.*^43^ reported that IL-6 was increased by the activation of the HPA axis, and Martos-Moreno *et al.*^44^ reported that secretion of IL-6 was inhibited by testosterone. As the 5HTTLPR S allele enhances both the HPA axis and testosterone reactivity^45^, the effects of 5HTTLPR on IL-6 levels may be negated by the antagonistic influences of HPA axis activity and testosterone. Additionally, ratings of subjective stress were not different across the 5HTTLPR genotypes. Similar null results in recent studies^46^,^47^ suggest that this genotype may not affect conscious and experienced sensitivity to acute stressors.

The present study has several limitations. First, the sample size was small; thus, the present findings are still preliminary and should be replicated with a larger sample. However, greater stress reactivity in pro-inflammatory cytokines, cardiovascular parameters and the HPA axis in SS allele carriers is consistent with many previous studies, which suggests the validity of the present findings. Additional findings of the association between 5HTTLPR and the IL-6/IL-10 balance in a larger sample size (Supplement) reiterate the reliability of the present findings. Second, in the present study, only male participants were examined to avoid variations in endocrine and immune parameters caused by the menstrual cycle in women. Bceause a previous study^35^ reported sex differences in the effects of 5HTTLPR on stress reactivity, the generalizability of the present findings need to be further examined using participants of both sexes. However, the Supplement shows no sex differences in the IL-6/IL-10 ratio between the SS and SL groups. Because Japanese male SS carriers show higher levels of trait anxiety than SL carriers, this association between 5HTTLPR genotype and anxiety is reversed in Japanese females^24^. In our study, trait anxiety was not different between the genotype groups, and thus, the present findings can be attributed to genotype but not to levels of trait anxiety. Moreover, gender and ethnicity interactions have been indicated for the association between 5HTTLPR genotype and the cerebrospinal fluid level of 5-hydroxyindoleacetic acid^48^. Thus, whether our findings are specific to Japanese males remains to be determined.

In conclusion, we clarified that Japanese male 5HTTLPR SS allele carriers show enhanced acute stress reactivity in terms of pro-inflammatory cytokine release as reflected by IL-1β, as well as the parameters of the endocrine and autonomic nervous systems, relative to SL carriers. The mechanisms explaining the effects of a polymorphism of a single gene on stress reactivity in such multi-level physiological systems remain unclear and awaits further research.

## Additional Information

**How to cite this article**: Yamakawa, K. *et al.* Serotonin transporter gene polymorphism modulates inflammatory cytokine responses during acute stress. *Sci. Rep.* **5**, 13852; doi: 10.1038/srep13852 (2015).

## Supplementary Material

Supplementary Figure

## Figures and Tables

**Table 1:** Participant characteristics.

	SS genotype	SL genotype
Age (years)	20.91 (2.11)	21.1 (2.17)
Height	171.36 (5.60)	171.50 (7.20)
Weight	66.36 (9.20)	63.80 (6.52)
BMI	22.62 (3.03)	21.83 (3.10)
STAI-trait	44.09 (10.14)	44.70 (5.50)

**Table 2:** Means (standard error of the mean) of the stress intensity and ANOVA results.

Group	Baseline	Task	Rest 30 min	Rest 60 min	Rest 90 min	Effect
Intensity of Stress (%)						
SS	29.33 (5.94)	57.22 (7.70)	22.67 (6.42)	22.44 (6.12)	20.22 (6.56)	*n.s.*
SL	28.89 (5.58)	57.44 (6.23)	28.67 (5.67)	31.33 (7.92)	24.00 (6.81)	

**Table 3:** Means (standard error of the mean) of IL-6 levels and ANOVA results.

Group	Baseline	Task	Rest 30 min	Rest 60 min	Rest 90 min	Effect
IL-6 (pg/ml)						
SS	2.51 (1.73)	3.08 (1.81)	3.13 (2.17)	2.56 (1.22)	3.51 (1.89)	*n.s.*
SL	1.55 (1.29)	2.42 (1.91)	3.13 (2.70)	2.60 (2.14)	2.95 (2.28)	

**Table 4:** Means (standard error of the mean) of the high frequency (HF) component and low frequency (LF)/HF ratio and ANOVA results.

Group	Baseline	Task 10 min	Task 15 min	Task 20 min	Rest 30 min	Rest 60 min	Rest 90 min	Effect
HF components (%)								
SS	0.57 (0.12)	0.43 (0.16)	0.34 (0.14)	0.33 (0.15)	0.42 (0.19)	0.38 (0.18)	0.38 (0.11)	*n.s.*
SL	0.38 (0.22)	0.37 (0.22)	0.28 (0.13)	0.29 (0.16)	0.38 (0.16)	0.34 (0.17)	0.31 (0.19)	
LF/ HF ratio								
SS	1.05 (0.47)	1.79 (0.69)	3.28 (1.68)	3.20 (2.24)	2.13 (0.87)	2.86 (1.77)	2.03 (0.80)	*n.s.*
SL	3.02 (2.77)	3.21 (2.34)	3.54 (1.92)	3.75 (2.83)	2.24 (0.96)	2.64 (1.01)	4.37 (4.02)	

## References

[b1] McEwen B. S. Stress, adaptation, and disease: Allostasis and allostatic load. Ann. N. Y. Acad. Sci. 840, 33–4 (1998). 9629234 10.1111/j.1749-6632.1998.tb09546.x

[b2] Charmandari E., Tsigos C. & Chrousos G. Endocrinology of the stress response. Ann. Rev. Physiol. 67, 259–284 (2005). 15709959 10.1146/annurev.physiol.67.040403.120816

[b3] Collier D. A., Stöber G. Li T., Heils A., Catalano M. & Di Bella D. A novel functional polymorphism within the promoter of the serotonin transporter gene: Possible role in susceptibility to affective disorders. Mol. Psychiatry. 1, 453–460 (1996). 9154246

[b4] Lesch K. P. *et al.* Association of anxiety-related traits with a polymorphism in the serotonin transporter gene regulatory region. Science. 274, 1527–1531 (1996). 8929413 10.1126/science.274.5292.1527

[b5] Bradley S. L., Dodelzon K., Sandhu H. K. & Philibert R. A. Relationship of serotonin transporter gene polymorphisms and haplotypes to mRNA transcription. Am. J. Med. Genet. B. Neuropsychiatr. Genet. 136, 58–61 (2005). 15858822 10.1002/ajmg.b.30185

[b6] Tjurmina O. A., Armando I., Saavedra J. M., Goldstein D. S. & Murphy D. L. Exaggerated adrenomedullary response to immobilization in mice with targeted disruption of the serotonin transporter gene. Endocrinology. 143, 4520–4526 (2002). 12446578 10.1210/en.2002-220416

[b7] Li Q. *et al.* Medial hypothalamic 5-hydroxytryptamine (5-HT)1A receptors regulate neuroendocrine responses to stress and exploratory locomotor activity: Application of recombinant adenovirus containing 5-HT1A sequences. J. Neurosci. 24, 10868–10877 (2004). 15574737 10.1523/JNEUROSCI.3223-04.2004 PMC6730203

[b8] Ohira H. *et al.* Polymorphism of the serotonin transporter gene modulates brain and physiological responses to acute stress in Japanese men. Stress. 12, 533–543 (2009). 19658029 10.3109/10253890902787826

[b9] Way B. M. & Taylor S. E. The serotonin transporter promoter polymorphism is associated with cortisol response to psychosocial stress. Biol. Psychiatry. 67, 487–492 (2010). 20006325 10.1016/j.biopsych.2009.10.021 PMC2824040

[b10] Gotlib I. H., Joormann J., Minor K. L. & Hallmayer J. HPA axis reactivity: a mechanism underlying the associations among 5-HTTLPR, stress, and depression. Biol. Psychiatry. 63, 847–851 (2008). 18005940 10.1016/j.biopsych.2007.10.008 PMC2373542

[b11] Brydon L. *et al.* Psychological stress activates interleukin-1beta gene expression in human mononuclear cells. Brain. Behav. Immun. 19, 540–546 (2005). 16214025 10.1016/j.bbi.2004.12.003

[b12] Steptoe A., Hamer M. & Chida Y. The effects of acute psychological stress on circulating inflammatory factors in humans: A review and meta-analysis. Brain Behav. Immun. 21, 901–912 (2007). 17475444 10.1016/j.bbi.2007.03.011

[b13] Baumann H. & Gauldie J. The acute phase response. Immunol. Today. 15, 74–80 (1994). 7512342 10.1016/0167-5699(94)90137-6

[b14] Black P. H. The inflammatory consequences of psychological stress: Relationship to insulin resistance, obesity, atherosclerosis and diabetes mellitus, type II. Med. Hypotheses. 67, 879–891 (2006). 16781084 10.1016/j.mehy.2006.04.008

[b15] Amaral W. Z., Lubach G. R., Bennett A. J. & Coe C. L. Inflammatory vulnerability associated with the rh5-HTTLPR genotype in juvenile rhesus monkeys. Genes Brain Behav. 12, 353–360 (2012). 23331374 10.1111/gbb.12023 PMC3618503

[b16] Fredericks C. A. *et al.* Healthy young women with serotonin transporter SS polymorphism show a pro-inflammatory bias under resting and stress conditions. Brain Behav. Immun. 24, 50–57 (2010). 10.1016/j.bbi.2009.10.014 PMC2826575 19883751

[b17] Yirmiya R., Winocur G. & Goshen I. Brain interleukin-1 is involved in spatial memory and passive avoidance conditioning. Neurobiol. Learn Mem. 78, 379–389 (2002). 12431424 10.1006/nlme.2002.4072

[b18] Heinz A. *et al.* Effects of acute psychological stress on adhesion molecules, interleukins and sex hormones: implications for coronary heart disease. Psychopharmacology (Berl) 165, 111–117 (2003). 12417965 10.1007/s00213-002-1244-6

[b19] Yamakawa K. *et al.* Transient responses of inflammatory cytokines in acute stress. Biol. Psychol. 82, 25–32 (2009). 19446599 10.1016/j.biopsycho.2009.05.001

[b20] Kimura K., Isowa T., Ohira T. & Murashima S. Temporal variation of acute stress responses in sympathetic nervous and immune systems. Biol. Psychol. 70, 131–139 (2005). 15908100 10.1016/j.biopsycho.2004.12.006

[b21] Kirschbaum C., Pirke K. M. & Hellhammer D. H. The ‘Trier Social Stress Test’—a tool for investigating psychobiological stress responses in a laboratory setting. Neuropsychobiology. 28, 76–81 (1993). 8255414 10.1159/000119004

[b22] Allen A. P., Kennedy P. J., Cryan J. F., Dinan T. G. & Clarke G. Biological and psychological markers of stress in humans: Focus on the Trier Social Stress Test. Neurosci Biobehav Rev. 38, 94–124 (2014). 24239854 10.1016/j.neubiorev.2013.11.005

[b23] Kudielka B. M. & Kirschbaum C. Sex differences in HPA axis responses to stress: A review. Biol. Psychol. 69, 113–132 (2005). 15740829 10.1016/j.biopsycho.2004.11.009

[b24] Mizuno T. *et al.* Gender difference in association between polymorphism of serotonin transporter gene regulatory region and anxiety. J. Psychosom. Res. 60, 91–97 (2006). 16380315 10.1016/j.jpsychores.2005.06.068

[b25] Nakazato K. & Mizuguchi T. Development and validation of Japanese version of State-Trait Anxiety Inventory. Shinshin-Igaku. 22, 107–112 (1982).

[b26] Speilberger C. D., Gorusch R. L. & Lushene R. D. STAI manual for the State-Trait Anxiety Inventory. Consulting Psychologists Press, Palo Alto, CA (1970).

[b27] Matsunaga M., Yamauchi T., Konagaya T., Nogimori T. & Ohira H. Psychological and physiological responses accompanying positive emotions elicited on seeing favorite persons. J. Posit. Psychol. 3, 192–201 (2008).

[b28] Xhyheri B., Manfrini O., Mazzolini M., Pizzi C. & Bugiardini R. Heart Rate. Variability Today. Appl. Psychophysiol Biofeedback. 55, 321–31 (2012). 10.1016/j.pcad.2012.09.001 23217437

[b29] Dickerson S. S. & Kemeny M. E. Acute stressors and cortisol responses: a theoretical integration and synthesis of laboratory research. Psychol. Bull. 130, 355–391 (2004). 15122924 10.1037/0033-2909.130.3.355

[b30] Jennings J. R. & Wood C. C. Letter: The epsilon-adjustment procedure for repeated-measures analyses of variance. Psychophysiology. 13, 277–278 (1976). 1273235 10.1111/j.1469-8986.1976.tb00116.x

[b31] Gavrilin M. A., Deucher M. F., Boeckman F. & Kolattukudy P. E. Monocyte chemotactic protein 1 upregulates IL-1β expression in human monocytes. Biochem. Biophys. Res. Commun. 14, 37–42 (2000). 11027635 10.1006/bbrc.2000.3619

[b32] Benschop R. J., Rodriguez-Feuerhahn M. & Schedlowski M. Catecholamine-induced leukocytosis: early observations, current research, and future directions. Brain Behav. Immun. 10, 71–91 (1996). 10.1006/brbi.1996.0009 8811932

[b33] Bosch J. A., Berntson G. B., Cacioppo J. T., Dhabhar F. S. & Marucha P. T. Acute stress evokes selective mobilization of T cells that differ in chemokine receptor expression: a potential pathway linking immunologic reactivity to cardiovascular disease. Brain Behav. Immun. 17, 251–259 (2003). 12831827 10.1016/s0889-1591(03)00054-0

[b34] Rubartelli A., Cozzolino F., Talio M. & Sitia R. A novel secretory pathway forinterleukin-1 beta, a protein lacking a signal sequence. EMBO J. 9, 1503–1510 (1990). 2328723 10.1002/j.1460-2075.1990.tb08268.x PMC551842

[b35] McCaffery J. M., Bleil M., Pogue-Geile M. F., Ferrell R. E. & Manuck S. B. Allelic variation in the serotonin transporter gene-linked polymorphic region (5-HTTLPR) and cardiovascular reactivity in young adult male and female twins of European-American descent. Psychosom. Med. 65, 721–728 (2003). 14508012 10.1097/01.psy.0000088585.67365.1d

[b36] Way B. M. & Taylor S. E. A polymorphism in the serotonin transporter gene moderates cardiovascular reactivity to psychosocial stress. Psychosom. Med. 73, 310–317 (2011). 21364196 10.1097/PSY.0b013e31821195ed PMC3090451

[b37] Barton D. A. *et al.* Elevated brain serotonin turnover in patients with depression: effect of genotype and therapy. Arch. Gen. Psychiatry. 65, 38–46 (2008). 18180427 10.1001/archgenpsychiatry.2007.11

[b38] Hariri A. R. & Holmes A. Genetics of emotional regulation: the role of the serotonin transporter in neural function. Trends Cogn. Sci. 10, 182–191 (2006). 16530463 10.1016/j.tics.2006.02.011

[b39] Marsland A. L., Cohen S., Rabin B. S. & Manuck S. B. Association between stress, trait negative affect, acute immune reactivity, and antibody responses to hepatitis B injection in healthy young adults. Health Psychol. 20, 4–11 (2001). 11199064

[b40] Isowa T., Ohira H. & Murashima S. Reactivity of immune, endocrine and cardiovascular parameters to active and passive acute stress. Biol. Psychol. 65, 101–120 (2004). 14706434 10.1016/s0301-0511(03)00115-7

[b41] Benschop R. J., Oostveen F. G., Heijnen C. J. & Ballieux R. E. Beta 2-adrenergic stimulation causes detachment of natural killer cells from cultured endothelium. Eur. J. Immunol. 23, 3242–3247 (1993). 8258340 10.1002/eji.1830231230

[b42] Cupps T. R. & Fauci A. S. Corticosteroid-mediated immunoregulation in man. Immunol. Rev. 65, 133–55 (1982). 6214495 10.1111/j.1600-065x.1982.tb00431.x

[b43] Zarković M. *et al.* Cortisol response to ACTH stimulation correlates with blood interleukin 6 concentration in healthy humans. Eur. J. Endocrinol. 159, 649–652 (2008). 18753312 10.1530/EJE-08-0544

[b44] Martos-Moreno G. A., Barrios V. & Argente J. Normative data for adiponectin, resistin, interleukin 6, and leptin/receptor ratio in a healthy Spanish pediatric population: relationship with sex steroids. Eur. J. Endocrinol. 155, 429–434 (2006). 16914597 10.1530/eje.1.02227

[b45] Tinajero J. C., Fabbri A. & Dufau M. L. Regulation of corticotropin-releasing factor secretion from Leydig cells by serotonin. Endocrinology. 130, 1780–1788 (1992). 1312425 10.1210/endo.130.4.1312425

[b46] Ohira H. *et al.* Imaging brain and immune association accompanying cognitive appraisal of an acute stressor. NeuroImage. 39, 500–514 (2008). 17913515 10.1016/j.neuroimage.2007.08.017

[b47] Cerit H., Jans L. A. & Van der Does W. The effect of tryptophan on the cortisol response to social stress is modulated by the 5-HTTLPR genotype. Psychoneuroendocrinology. 38, 201–208 (2012). 22717170 10.1016/j.psyneuen.2012.05.016

[b48] Williams R. B. *et al.* Serotonin-related gene polymorphisms and central nervous system serotonin function. Neuropsychopharmacology. 28, 533–541 (2003). 12629534 10.1038/sj.npp.1300054

