# Supplementary material for: Serotonin transporter gene polymorphism modulates inflammatory cytokine responses during acute stress
Source: Sci Rep. 2015 Sep 9;5:13852. doi: 10.1038/srep13852 (PMC4563370; doi:10.1038/srep13852)
Supplement: Supplementary Figure [file srep13852-s1.pdf]

Serotonin transporter gene polymorphism modulates inflammatory cytokine responses during acute stress

Kaori Yamakawa[a,b], Masahiro Matsunaga[c], Tokiko Isowa[d], Hideki Ohira[a]

[a]Department of Psychology, Graduate School of Environmental Studies, Nagoya University, Aichi, Japan

[b]Department of Psychology, School of Humanities, Tokaigakuen University, Aichi, Japan

[c]Department of Health and Psychosocial Medicine, School of Medicine, Aichi Medical University, Aichi, Japan

[d] School of Nursing, Faculty of Medicine, Mie University, Mie, Japan

Conflict of Interest Statement

All authors declare that there are no conflicts of interest.

*Correspondence should be addressed to Kaori Yamakawa

Department of Psychology, School of Humanities, Tokaigakuen University, 2-901, Nakahira, Tenpaku-ku, Aichi, 468-8514, Japan

Phone: +81 52-801-1201 E-mail: kaori.yamakaw@gmail.com

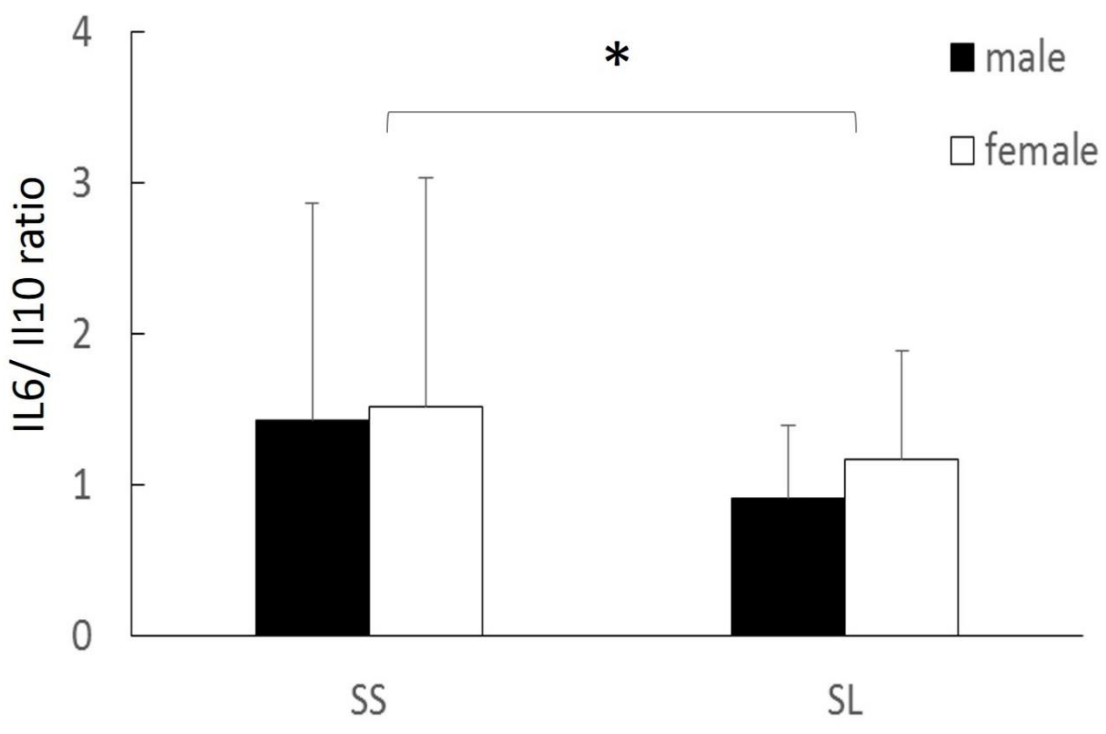

IL6/ Il10 ratio
4
3
2
1
0
*
male
female
SS
SL
